# Complete Ear Reconstruction With Pre-laminated Radial Artery-Based Free Flap in Old Traumatic Amputation

**DOI:** 10.7759/cureus.113463

**Published:** 2026-07-27

**Authors:** Aditya Aggarwal, Vimalendu Brajesh, Sukhdeep Singh, P I Pragyan Pratik, Sneha Sharma

**Affiliations:** 1 Plastic and Reconstructive Surgery, Medanta, Gurugram, IND

**Keywords:** case report, ear reconstruction, forearm free flap, prefabrication, prelamination, total ear amputation

## Abstract

Total ear reconstruction in old avulsion injuries can be extremely challenging for reconstructive surgeons due to regional scarring. This rules out the use of local flaps. In this case report, a young woman presented to us with a seven-year-old total amputation of the left ear, which was initially reconstructed with a Medpor implant and occipito-parietal fascial flap. The implant had to be later explanted due to infection. The authors decided to use a pre-laminated radial free flap with a costal cartilage graft framework for ear reconstruction.

## Introduction

The external ear is a pivotal component of the aesthetic balance of the face. Automobile accidents, violence and thermal injuries are the most common aetiologies for acquired deformities of the pinna [1]. Total ear reconstruction in cases of old avulsion injuries is a challenge for the reconstructive surgeon, given the paucity of local tissue available for cover.

Since the advent of total ear reconstruction by Schmieden in 1908, various methods for autologous costochondral cartilage or implant-based ear reconstruction have been reported in the literature, which include the use of postauricular skin, postauricular fascia, free/pedicled temporo-parietal fascia, and pre-laminated composite flaps like radial artery-based forearm free flap [2-4]. However, in old avulsion injuries, post-traumatic scarring around the auricular region renders the skin ineffective for draping an ear framework, thus ruling out the use of local flaps for reconstruction [4].

In this case report, due to extensive scarring of local tissue, the authors decided to proceed with a pre-laminated radial forearm free flap. This method has been used relatively rarely for ear reconstruction due to its complexity. In this technique, a costal cartilage framework is temporarily parked in the forearm, followed by a microsurgical transfer of the composite free flap to the desired position [5].

## Case presentation

Patient information

A 23-year-old female patient suffered from traumatic avulsion of the left ear, scalp, left cheek, left eye and a portion of the left orbit and skull in July 2011. She had since undergone multiple facial surgeries. Before presenting to us, she had already undergone reconstruction of her left ear with a Medpor implant with occipito-parietal fascial flap. The implant later got infected and had to undergo implant explantation. She had also undergone a left cranium reconstruction with a titanium mesh and anterolateral thigh free flap followed by its de-fattening. She also underwent the creation of the left auditory canal with split-thickness skin graft, left eye ptosis surgery, placement of an ocular implant, lateral canthal tendon creation, and multiple scar revisions. The patient was left hand-dominant and did not have any known co-morbidities.

Clinical findings

**Figure 1:**
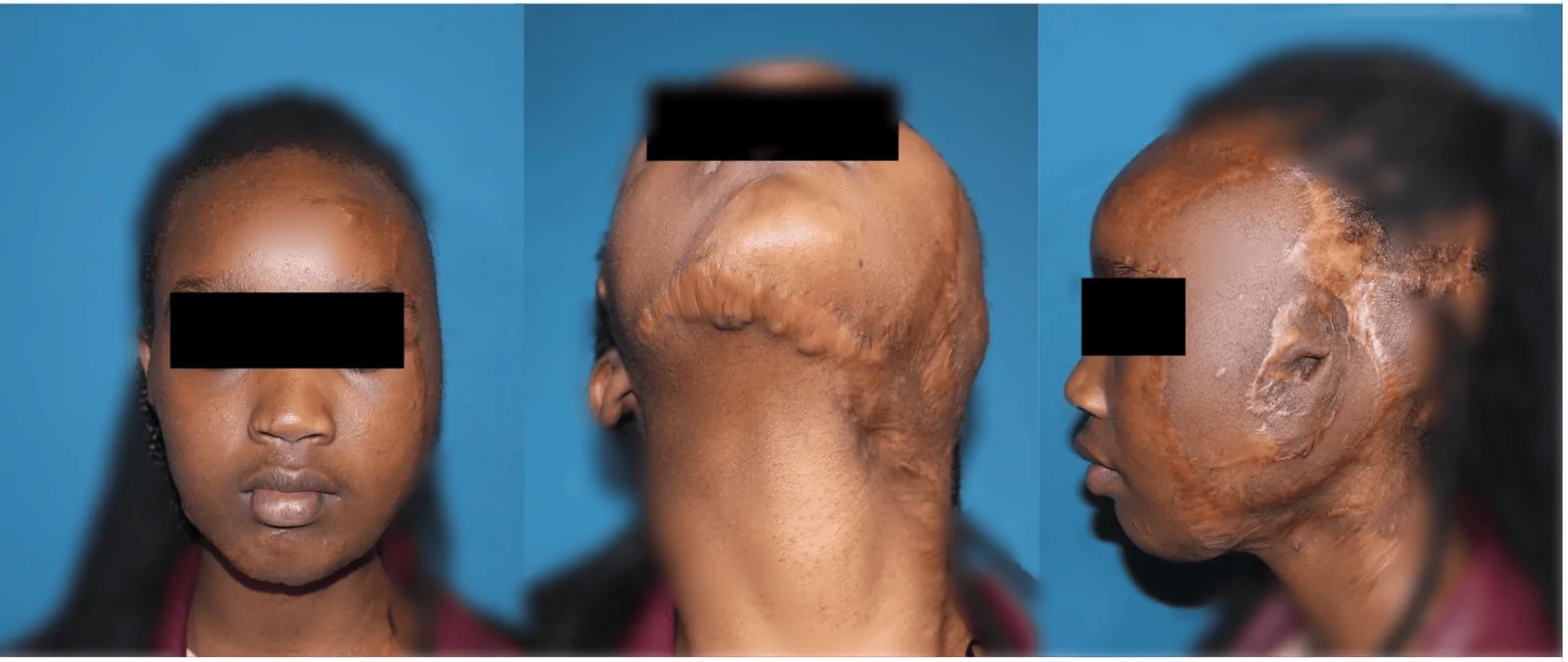
Pre-operative images

The patient presented to us for left ear reconstruction. On examination, the patient had an absent left ear with a patent external auditory canal, multiple facial scars with a well-settled free flap over the left side of the face, and scalp scarring with alopecia present on the left temporal region (Figure 1).

Timeline

A staged procedure was planned for reconstructing the left ear using a pre-laminated radial artery forearm free flap.

Stage 1: Ear Framework Creation and Pre-lamination of the Radial Artery Forearm Free Flap With the Ear Framework

**Figure 2:**
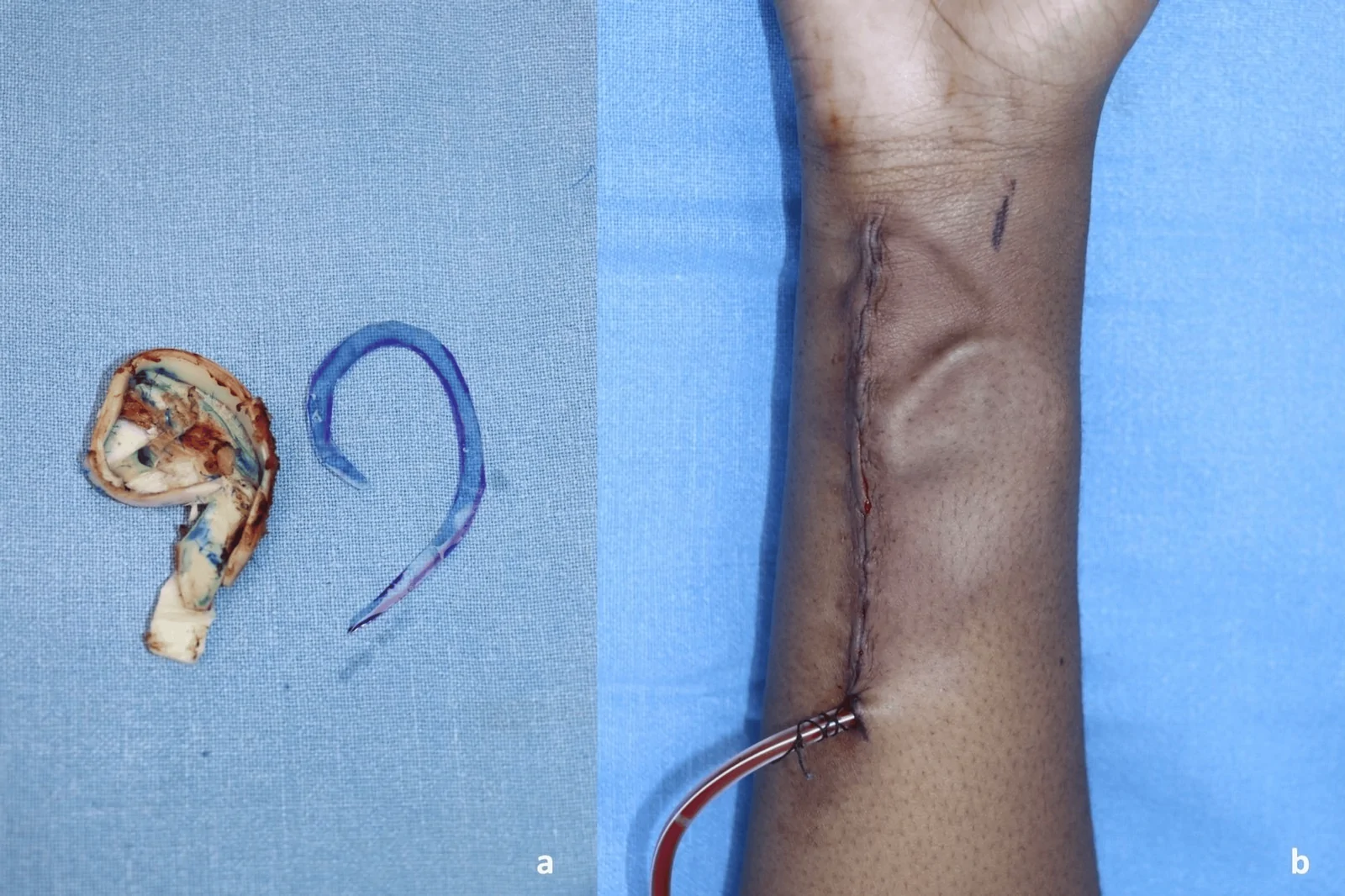
(a) Cartilage framework of the left ear constructed using autologous 6th, 7th, and 8th costochondral cartilage; (b) Final appearance of the ear framework positioned subcutaneously in the forearm pocket.

A cartilage block was harvested from the contralateral 6th-8th costal synchondrosis after placing a pre-designed ear template over it. The 6th and 7th costal cartilage was used for constructing the concha and antihelix-antitragus complex. The 8th costal cartilage was used for constructing the helical rim (Figure 2a). Under tourniquet control, an incision was made on the ulnar side of the volar surface of the distal right forearm, and a subcutaneous pocket was created measuring 4 cm in width and 8 cm in length. The cartilage framework measuring 3 cm in width and 4.6 cm in length was placed in this pocket with overlying skin flap thickness measuring 12 mm. A suction drain was inserted in the pocket to create negative pressure and reinforce the flap onto the shape of the underlying framework (Figure 2b).

Four expanders were inserted in the same sitting over various areas of the face for achieving skin expansion for use in scar revision at a later date and also for use as a full-thickness skin graft. Markings were done over previous facial scars. One large 300 cc expander was placed in the subgaleal plane in the occipital region; the second expander (100 cc) in the forehead. Another 50 cc expander was placed in the left cheek, and one 250 cc expander was placed in the left neck in the subplatysmal plane.

Stage 2: Flap Thinning

Three months later, thinning of the skin flap covering the cartilage framework was done to minimize subcutaneous tissue thickness over the cartilaginous framework and provide a better contour to the reconstructed ear. The entry point was the previous incision and the skin flap above the cartilaginous framework was raised in the subcutaneous plane and defatted to provide a well-vascularised skin flap of around 2 mm thickness.

Stage 3: Flap Delay and Prelamination of the Free Radial Artery Forearm Flap With Full Thickness Skin Graft

**Figure 3:**
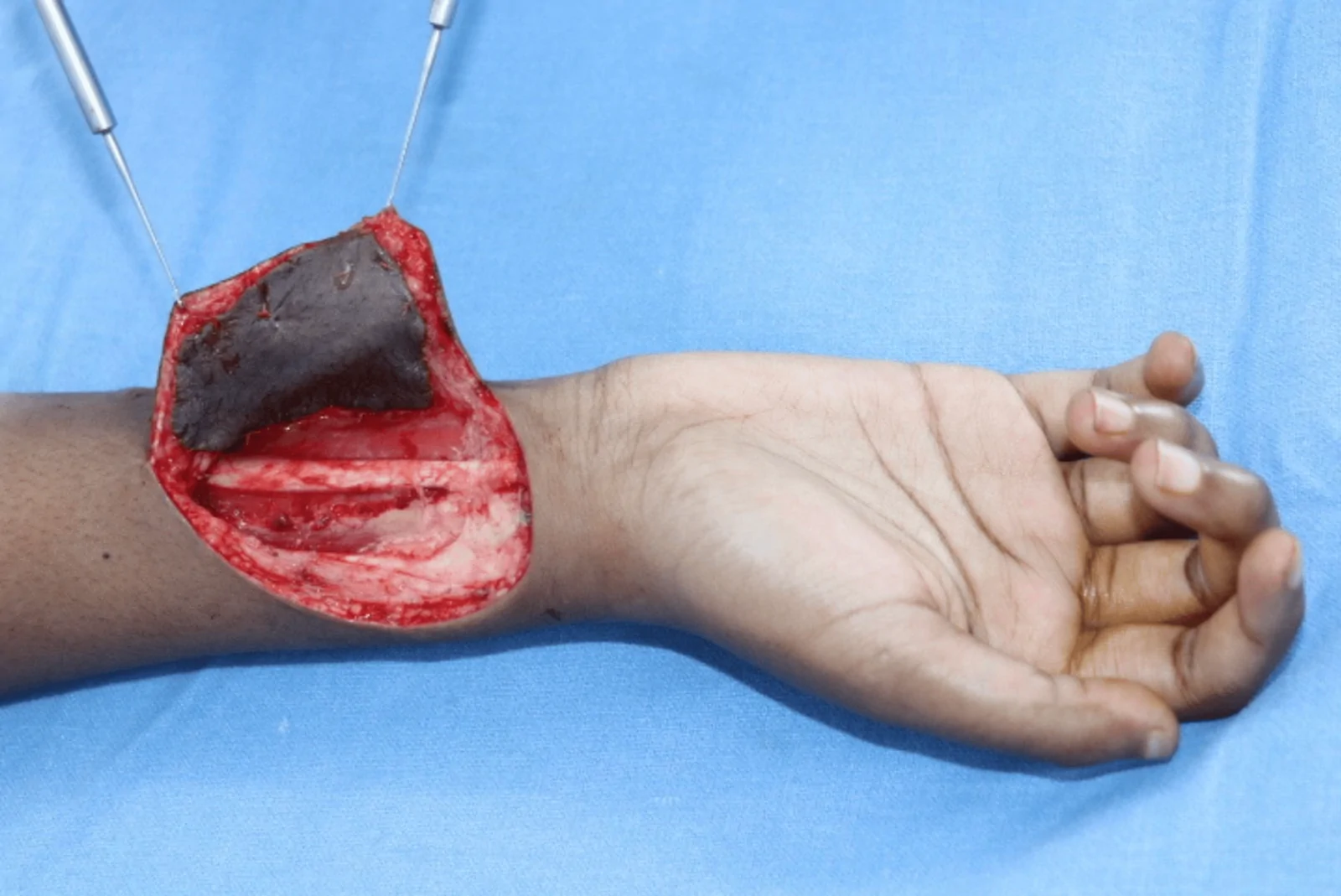
Pre-lamination of the ear framework using a full-thickness skin graft.

Four months later, an incision was made over the radial side of the flap, and the flap was raised along with the ear framework, keeping it attached over the ulnar aspect. The radial artery was identified and ligated to delay the flap. A full-thickness skin graft harvested from tissue-expanded excess skin of the temporal scalp was sutured to the fascial surface of the flap (Figure 3). A silicone sheet cut to the size of the flap was placed at the base of the flap, and the flap was sutured back to its original position.

Stage 4: Flap Islanding

**Figure 4:**
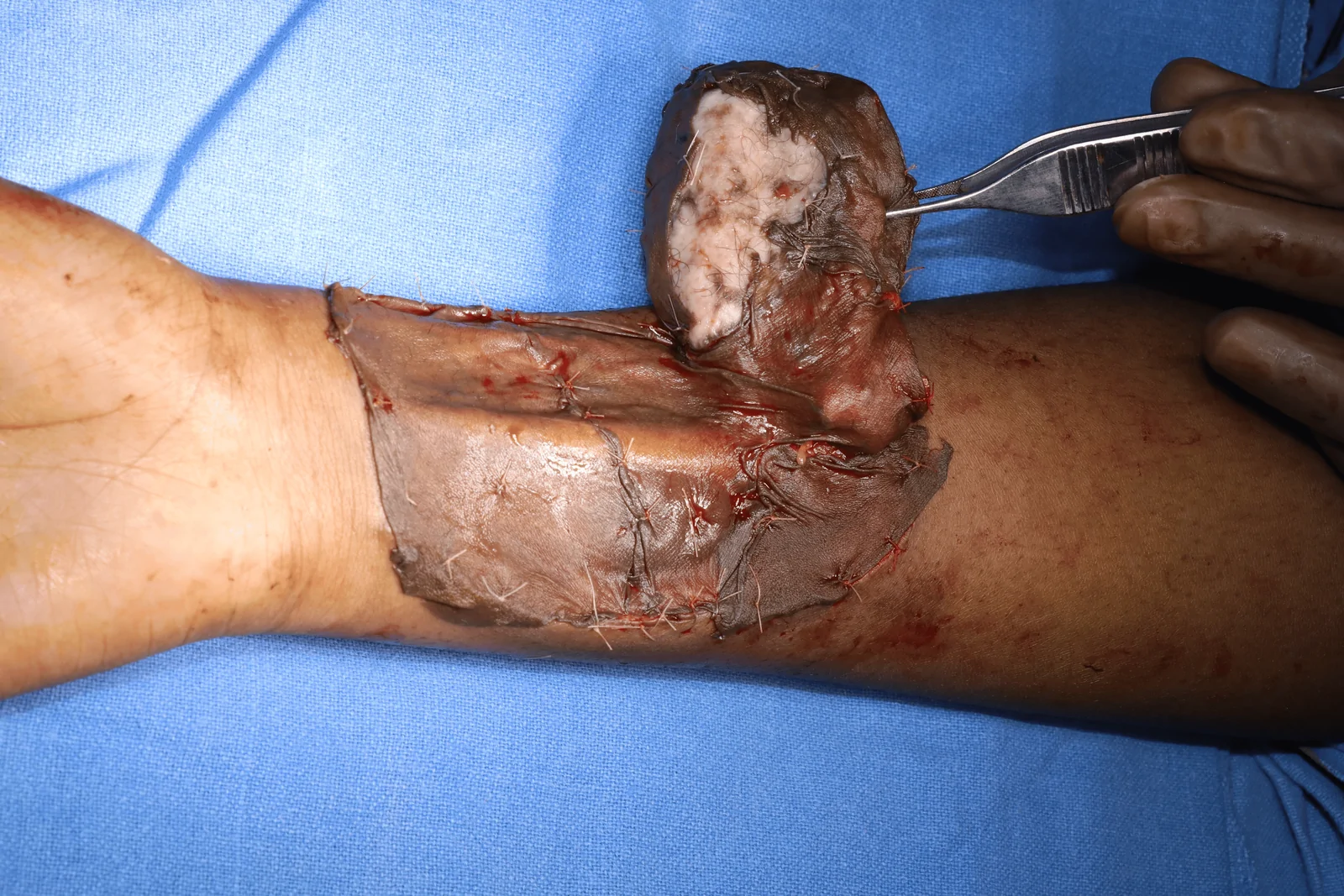
Islanding of the composite free radial artery forearm flap.

Five months later, islanding of the flap was performed, which remained pedicled at its proximal pole. The silicone sheet underneath it was removed, and the pseudo-capsule was excised. A split-thickness skin graft (STSG) harvested from the contralateral thigh was used to cover the raw volar surface of the forearm except for a small area around the pedicle. STSG was also used to cover the raw edges of the flap created after islanding it (Figure 4).

Stage 5: Final Flap Transfer

**Figure 5:**
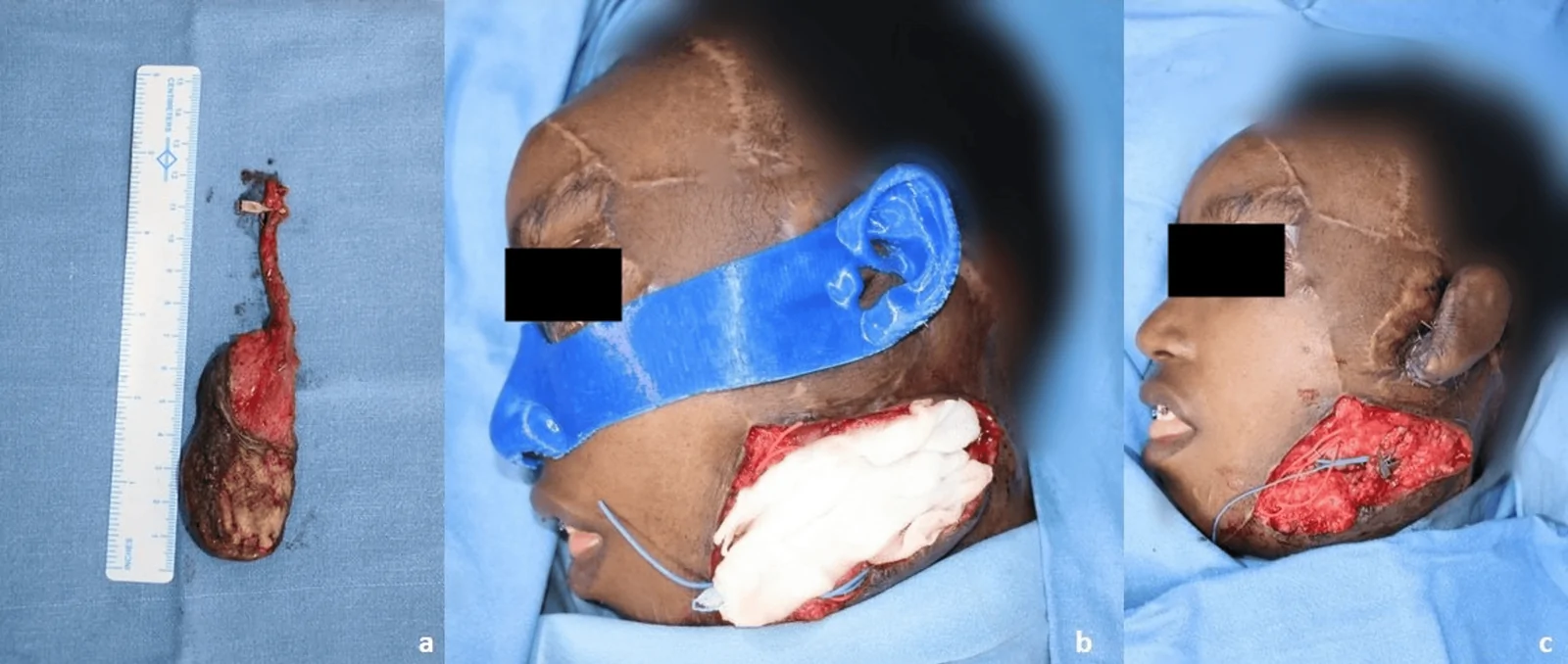
(a) Medial surface of composite free forearm flap with pedicle; (b) Positioning of the new ear using 3D-printed template; (c) Final intra-operative appearance.

Six and a half months later, the radial artery territory was identified using a hand-held Doppler and marked. The radial artery pedicle was identified and dissected retrogradely up to the flap. Pre-laminated multilayered auricular cartilage framework was elevated along with the radial artery pedicle (Figure 5a). At the recipient site, superficial temporal artery and two veins in the vicinity were identified and dissected. The positioning of the ear was done using a 3D-printed template created using virtual surgical planning using the computed tomography scan of the face with 0.6-0.7 mm cuts (Figure 5b). A subcutaneous tunnel was created for the passage of the flap pedicle from the region of the ear placement to the recipient vessels. The flap pedicle was ligated in the forearm and divided. Flap insetting was completed, and the pedicle passed through the subcutaneous tunnel. Anastomosis of the radial artery and two venae comitantes was done with the superficial temporal artery and the accompanying veins, respectively (Figure 5c).

Follow-up and outcomes

**Figure 6:**
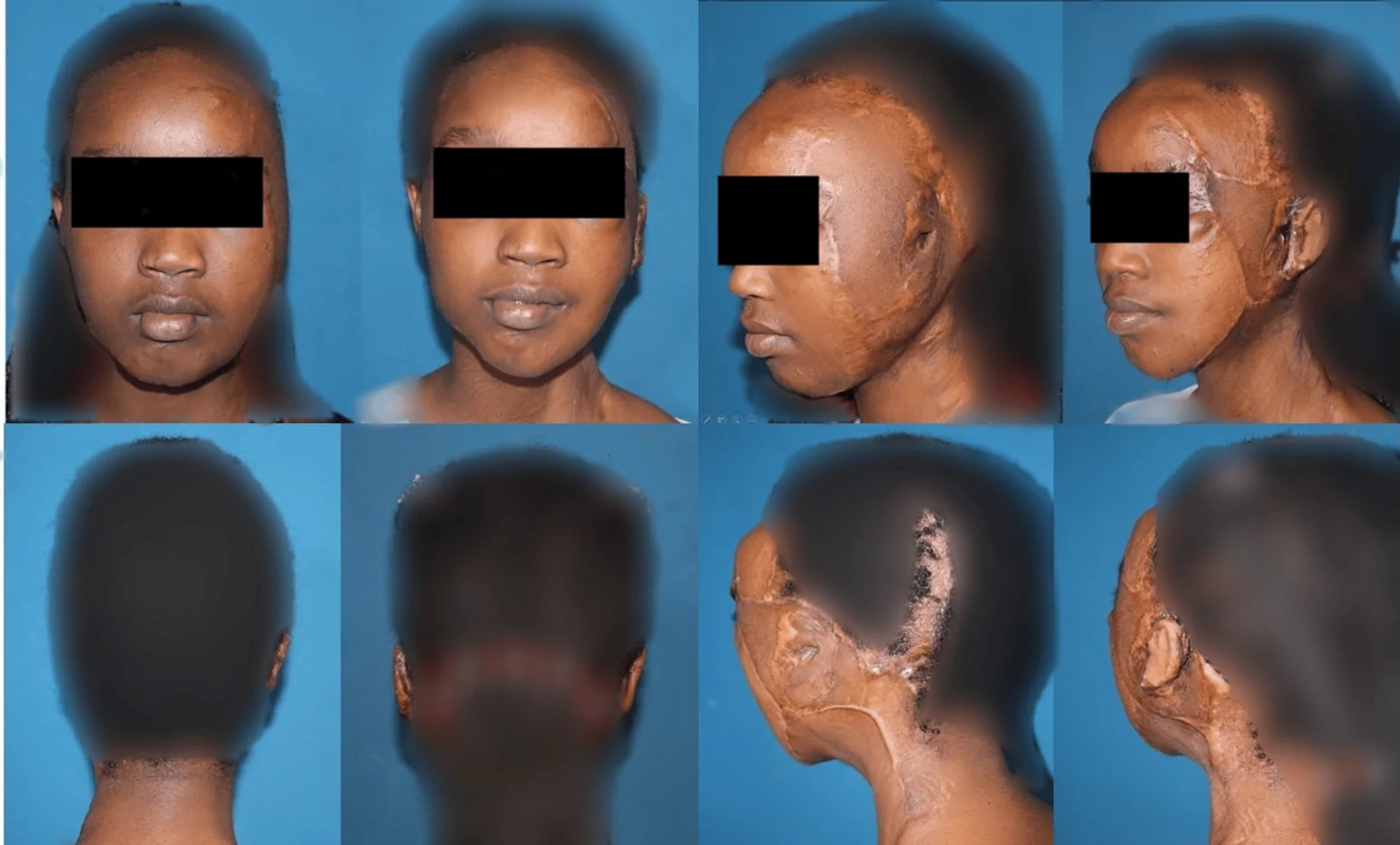
Comparison of pre-operative and post-operative images at 6-month follow-up.

**Figure 7:**
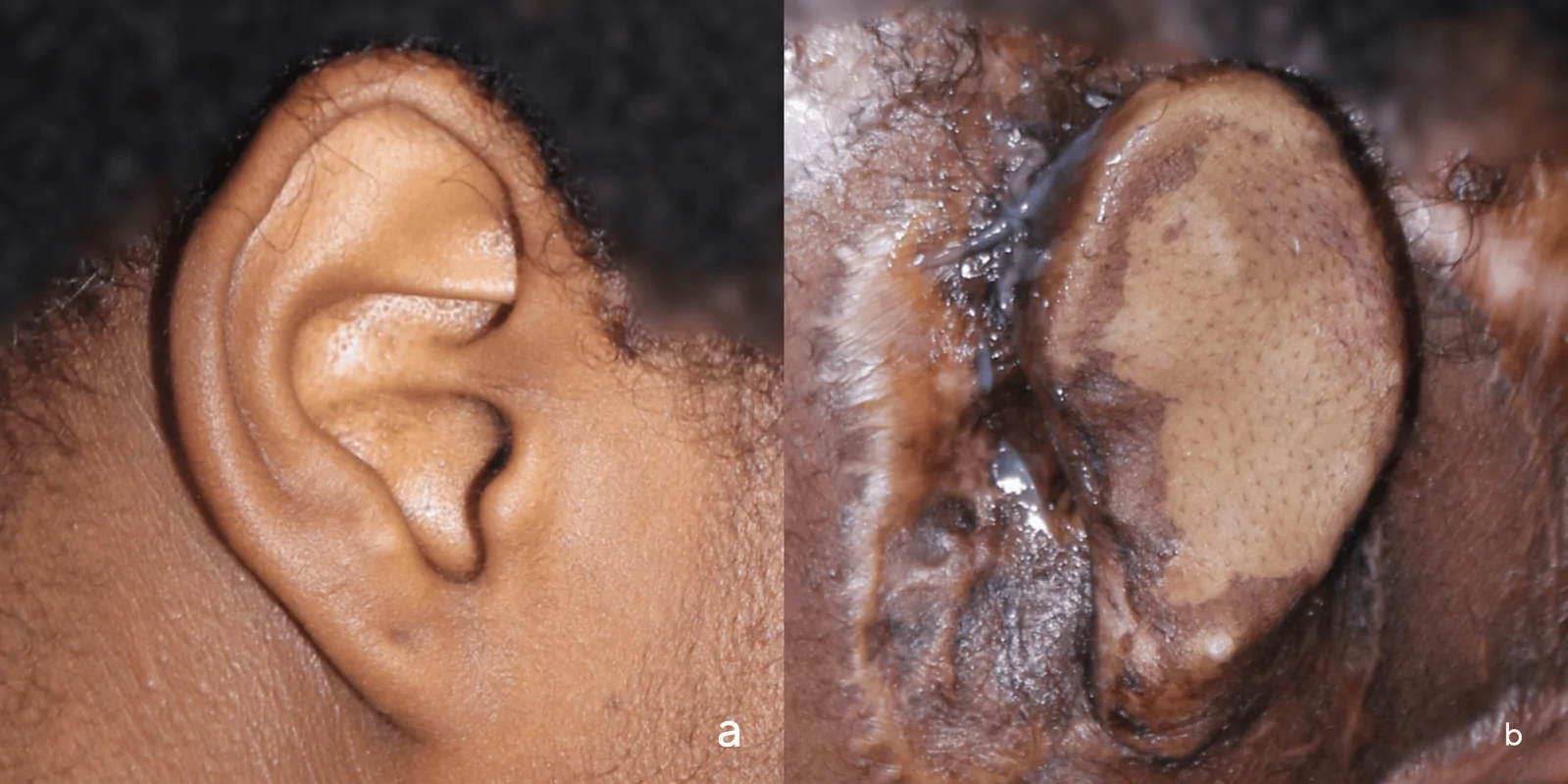
(a) Right ear for comparison; (b) Post operative image of total left ear reconstruction

The patient was followed up regularly for postoperative care. On POD 13, the suture line at the helix region was found to be dehisced along with minimal discharge. Under antibiotic coverage, the raw area was debrided, and the wound was closed. She continued to have an uneventful post-operative course. Dimensions of the reconstructed ear were similar to the contralateral healthy ear (Figures 6, 7).

Patient perspective

The patient was satisfied with her ear reconstruction due to regaining anatomical function such as wearing glasses, hearing aid and face mask.

## Discussion

Old avulsion injuries of the ear can be extremely challenging to manage, especially when combined with simultaneous multiple facial scar corrections. This is because regional skin is often scarred, tight, and fragile [4]. Moreover, simultaneous scar correction procedures prolonged the timeline of our ear reconstruction, making it a six-staged procedure.

In 1991, Sucur et al. first successfully devised the method of using a composite forearm free flap for total ear reconstruction [6]. Following which Zhou et al. in 1994 reported the use of this method in three cases of burn and avulsion injuries to the ear [7]. Both the authors agree on indications for this method, which include but are not limited to the unavailability of normal regional skin and tissue.

The best material to create the structural framework of the ear has always been a subject of debate. Synthetic materials like Medpor have been widely used [8]. In this case, the patient had undergone ear reconstruction using a Medpor implant seven years back, which was later explanted due to infection. Lower infection rates have been reported in autologous grafts as compared to Medpor implants [9]. Therefore, instead of repeating the earlier method, the authors decided to use the autologous costochondral cartilage as the patient was young and there were no contraindications for the same.

The use of the free radial forearm fasciocutaneous flap for ear reconstruction has been reported in the literature, although it remains relatively uncommon. This is mainly because of the thickness of forearm skin, which results in inadequate ear contour [7]. The main advantage of this flap is its long pedicle, which allows anastomosis with available vessels in the neck, without the need for a vein graft [7]. Due to the tensile strength of forearm skin, cartilage extrusion is rarely seen, and thus it is more suitable for covering stiff implants as compared to temporo-parietal fascia [10]. Whereas flap thinning may sometimes be required to provide adequate contour to the flap [11].

In this case, the reconstructed ear is well placed and provides a symmetry to the face which was previously missing. The patient was satisfied with the reconstruction.

The limitation in this case is the somewhat bulky appearance of the reconstructed ear. Further procedures like tragal construction can be undertaken to provide more definition.

## Conclusions

A pre-laminated composite radial artery forearm free flap with autologous costochondral cartilage is a creative approach for patients with old avulsion amputation of the ear with absent temporoparietal fascia or unhealthy skin around the auricular region. Although the reconstruction may initially appear bulky, a limitation that can be addressed with further staged procedures, this technique successfully restores facial symmetry and achieves patient satisfaction.
